# Students’ Beliefs About Trigger Warnings

**DOI:** 10.1177/00332941241308788

**Published:** 2024-12-13

**Authors:** A. Timur Sevincer, Leonie Tenbrueggen, Marvin Sokolis

**Affiliations:** 27720 Leuphana University Lueneburg, Lueneburg, Germany; Hanse-Wissenschaftskolleg Delmenhorst, Institute for Advanced Study, Delmenhorst, Germany; 27720 Leuphana University Lueneburg, Lueneburg, Germany

**Keywords:** Trigger warnings, student beliefs, attitudes, emotional reaction, avoidance

## Abstract

Trigger warnings aim to help people emotionally prepare for potentially disturbing material or avoid the material altogether. There has been a lively debate in society and academia whether the widespread use of trigger warnings helps, harms, or has no substantial impact. Recent meta-analytic evidence suggests trigger warnings have no effect on people’s emotional reaction, avoidance, and comprehension. They do however heighten a negative anticipatory reaction. We examined students’ attitudes toward trigger warnings in a non-English-speaking country – Germany, and whether their beliefs about the effects of trigger warnings on themselves and others match the meta-analytic evidence. Students held relatively positive attitudes toward trigger warnings and advocated their use. Their beliefs about the effects of trigger warnings however did not concur well with the actual effects. Our findings suggest that making students aware of the empirical evidence on trigger warnings would benefit discussions around trigger warnings.

Trigger warnings, content warnings, or content notes are information about upcoming material that can be emotionally disturbing (Bellet et al., 2018). Originally, such warnings have been introduced to prevent people who suffer from traumatic experiences from reexperiencing aspects of the trauma (e.g., flashback memories) by being suddenly confronted with reminders of the traumatic events (Boysen et al., 2017). Over time however, the use of warnings expanded to prevent anyone, not just trauma survivors, from experiencing discomfort (Bellet et al., 2018). Trigger warnings thus have two main functions: First, allowing people to emotionally prepare for the material to reduce their negative emotional response, and second, allowing people to avoid the material altogether (Bridgland et al., 2023).

Although trigger warnings are frequently used in higher education – over half of U.S. professors reported having used them (Kamenetz, 2016) – there is a lively debate about their usefulness. Article headlines in widely circulated newspapers read: “Warning: the literary canon could make students squirm” - The New York Times (Medina, 2014), and “What if Trigger warnings don’t work?” - The New Yorker (Gersen, 2021). This debate is also evident in online forums. A content-analysis of over 1500 online comments found that pro-warnings arguments were protection of mental health, promotion of higher empathic concern, and conveying positive values. Contra-warnings arguments were infantilization of students, restriction of academic freedom, and unfair distribution of responsibility on the lecturers (George & Hovey, 2020). Whereas lecturers’ attitudes toward warnings were rather negative, students’ attitudes were more positive (Bentley, 2017; Beverly et al., 2018).

In addition to the debate in education there is also a debate in psychology about whether trigger warnings actually have the desired effects of reducing a negative reaction and leading people to avoid the material. A recent meta-analysis (Bridgland et al., 2023) involving twelve experimental studies found that warnings *did not* reduce people’s negative emotional reaction to the material or lead them to avoid the material. Rather, warnings *increased* a negative anticipatory response after the warning was given but *before* the material was presented (e.g., Boysen et al., 2021; Bridgland & Takarangi, 2021). Trigger warnings also *did not* facilitate the comprehension of the material (measured by e.g. multiple-choice tests on the content; Boysen et al., 2021). The meta-analysis included many studies with trauma survivors and the effects held for this group. Other studies found that warnings even *increased* an immediate anxiety response to the material in people who met the clinical cut-off for Post-Traumatic-Stress-Disorder (Jones et al., 2020). Studies such as these led the Association for Psychological Science to conclude in one report that “Caution: content warnings do not reduce distress, study shows” (Association for Psychological Science, 2023), and “trigger warnings fail to help and may even harm” (Association for Psychological Science, 2020). Despite this growing body of evidence that warnings do not work some students may still request their use.

## The Present Research

We had two aims: First, because research on trigger warnings was almost exclusively done in the U.S. (and UK), we examined the prevalence of warnings and students’ attitudes toward them in a non-English-speaking country – Germany, to examine whether the use of warnings has spread to other countries than the U.S. (and UK).

Second, even though empirical evidence suggests trigger warnings may not work, students hold positive attitudes toward their use (Bentley, 2017). We therefore wondered how accurate students’ beliefs about the effects of the warnings are. Are students in favor of warnings because they (mistakenly) believe warnings have the desired effects to reduce a negative reaction and lead people to avoid material? And are they aware that warnings may even *increase* anticipatory anxiety?

To examine students’ beliefs about trigger warnings and contrast them with the actual empirical evidence, we asked students to report their beliefs about the effects of warnings in the four domains examined in the meta-analysis by Bridgland et al. (2023): Response affect, avoidance, anticipatory affect, and comprehension. Because people see others as more vulnerable to negative events than themselves (Perloff & Fetzer, 1986), and students may advocate trigger warnings out of concern for others rather than themselves, we asked them to report their beliefs about the effects of warnings on themselves and on others.

## Method

### Participants

We recruited 205 German students from Germany (121 female, 72 male, 8 diverse, 5 unidentified, *M*_age_ = 25.9 years, *SD* = 8.0). The study was advertised on Prolific as “a study on lecture contents at universities”. To obscure the nature of the study and avoid that students with strong opinions about trigger warnings disproportionally self-select for taking part in the study, we did not use the term trigger warnings in the description of the study on Prolific. Using the prescreening option, participants had to be currently enrolled at a German university in a subject where we deemed trigger warnings might occur (e.g., psychology, humanities; see Supplemental Table S1). Participants received €1,30 for taking part. Because the pool of participants fulfilling our criteria on Prolific was limited (only 508), we aimed to recruit 200 participants. Sensitivity analysis yielded that with this sample size we could detect a difference between two dependent means of *d* = 0.23 with 95% power. The preregistration, data, code, and complete materials are available at: https://shorturl.at/L8zT6.

### Procedure

On the first page of the online questionnaire, participants were informed that they would be asked several questions about their attitudes towards the content of university lectures. They were told that participation was voluntary and that they could withdraw at any time. On the next page, they were given a definition of trigger warning adapted from the definition by Bridgland et al. (2023): “Trigger warnings, content warnings or content notes are warnings of upcoming content that may contain themes related to past negative experiences. They are intended to help people prepare for or comletely avoid upcoming content.”

#### Prevalence

We used three items. “Did you ever experience that students requested trigger warnings in courses?”, “Have you ever discussed the topic of trigger warnings with other students or lecturers?” (4-point scales, 1 = *no, never*, 2 = *yes, one or two times*, 3 = *yes three to five times*, 4 = *yes, more than five times*), and “What percentage of your courses do you estimate had trigger warnings?” (open ended). The first item was adapted from a survey on trigger warnings (National Coalition Against Censorship, 2015) and translated into German language by us, the others were generated in German language by ourselves.

#### Attitudes

We used five items (Table 1) from the General Attitude Towards Trigger Warnings Scale (Cares et al., 2018; 6-point scale; 0 = *strongly disagree*, 5 = *strongly agree*). We omitted two items^1^ from the original scale because they were difficult to apply to Germany. Internal consistency of the remaining five items was α = .78. We reverse-coded the items 1, 2, and 3 (Table 1) so that higher numbers indicate more positive attitudes. We then averaged all five items into one general attitude towards trigger warnings index.

**Table 1.** Ms, SDs, and One-Sample T-Tests on the Scale Midpoint (2.5 of the 5-Point-scale, 0 = Strongly Disagree, 5 = Strongly Agree) for Each Item of the General Attitude Toward Trigger Warnings Scale.

Item	*M* (*SD)*	*df*	*t*	*p*	95% CI	*d*
1 Trigger warnings are unnecessary	1.17 (1.34)	197	14.05	<.001	[−1.52, −1.15]	1.00
2 Students who need trigger warnings should not be taking classes that talk about sensitive or offensive material	1.85 (1.61)	197	5.65	<.001	[−0.87, −0.42]	0.40
3 Students who need trigger warnings are too coddled	1.40 (1.40)	197	11.10	<.001	[−1.30, −0.91]	0.79
4 All classes should give trigger warnings on the syllabus, regardless of topic	1.43 (1.44)	197	10.44	<.001	[−1.27, −0.86]	0.74
5 This university should require professors to put a trigger warning on the syllabus	2.40 (1.53)	197	0.93	.355	[−0.32, 0.11]	0.07

We also used the Trigger Warnings Attitudes Assessment (Bellet et al., 2018). Participants are asked: “Do you think that trigger warnings should be used?” (1 = *yes*, 2 = *no*). If the respond yes, they are asked “Why do you think that trigger warnings should be used?” and are given a list of six possible reasons (e.g., “offensive material can cause psychological harm to everyone”). They could also mention reasons that were not listed.

#### Beliefs

We used a total of eight bipolar items: We created two items to assess what students think about the effects of trigger warnings on them and others, respectively, for each of the four domains from Bridgland et al. (2023).

#### Response Affect

We asked whether trigger warnings would change one’s own reaction, and the reaction of others, respectively: “Do you think that trigger warnings change the negative reaction [of yourself/others], while consuming the contents?” (9-point scale, −4 = *strongly decrease*, −2 = *somewhat decrease*, 0 = *no influence*, 2 = *somewhat increase*, 4 = *strongly increase*).

#### Avoidance

“Do you think that trigger warnings will cause [yourself/others] to avoid content?” (9-point scale, −4 = *definitely avoid*, −2 = *rather avoid*, 0 = *no influence*, 2 = *rather approach*, 4 = *definitely approach*).

#### Anticipatory Affect

“Do you think that trigger warnings will elicit negative emotional reactions [in yourself/others] *before* consuming the contents?” (9-point scale, −4 = *strongly disagree*, −2 = *disagree*, 0 = *neither nor*, 2 = *agree*, 4 = *strongly agree*).

#### Comprehension

“Do you think that trigger warnings change the comprehension of the material [for yourself/others]?” (9-point scale, −4 = *strongly impair*, −2 = *somewhat impair*, 0 = *no influence*, 2 = *somewhat improve*, 4 = *strongly improve*).

#### Demographics

Participants reported their age, gender, major subject, left-right political orientation (“What is your political orientation?”; 11-point scale, 0 = *extreme left*, 6 = *cente*r, 11 = *extreme right*; Kroh, 2007), and whether they were familiar with the meta-analysis by Bridgland et al. (2023). They were fully debriefed.

## Results

Following our preregistration, we excluded seven participants because they reported being familiar with the meta-analysis by Bridgland et al. (2023).

### Prevalence

The majority of students reported never having witnessed students demanding trigger warnings (60%) or having discussed the topic themselves (61%). A substantial minority witnessed it (34%) or discussed the topic (26%) once or twice. A small fraction witnessed (4%) or discussed (8%) it three to five times and an even smaller fraction witnessed (2%) or discussed (5%) it more than five times.

We dummy-coded students’ responses on the open-ended question how many of their courses contained warnings. Forty-two percent reported warnings in none of their courses, 39% in 1–10%, 14% in 11–30%, and 5% in over 30% of their courses.

### Attitudes

**Figure 1.**
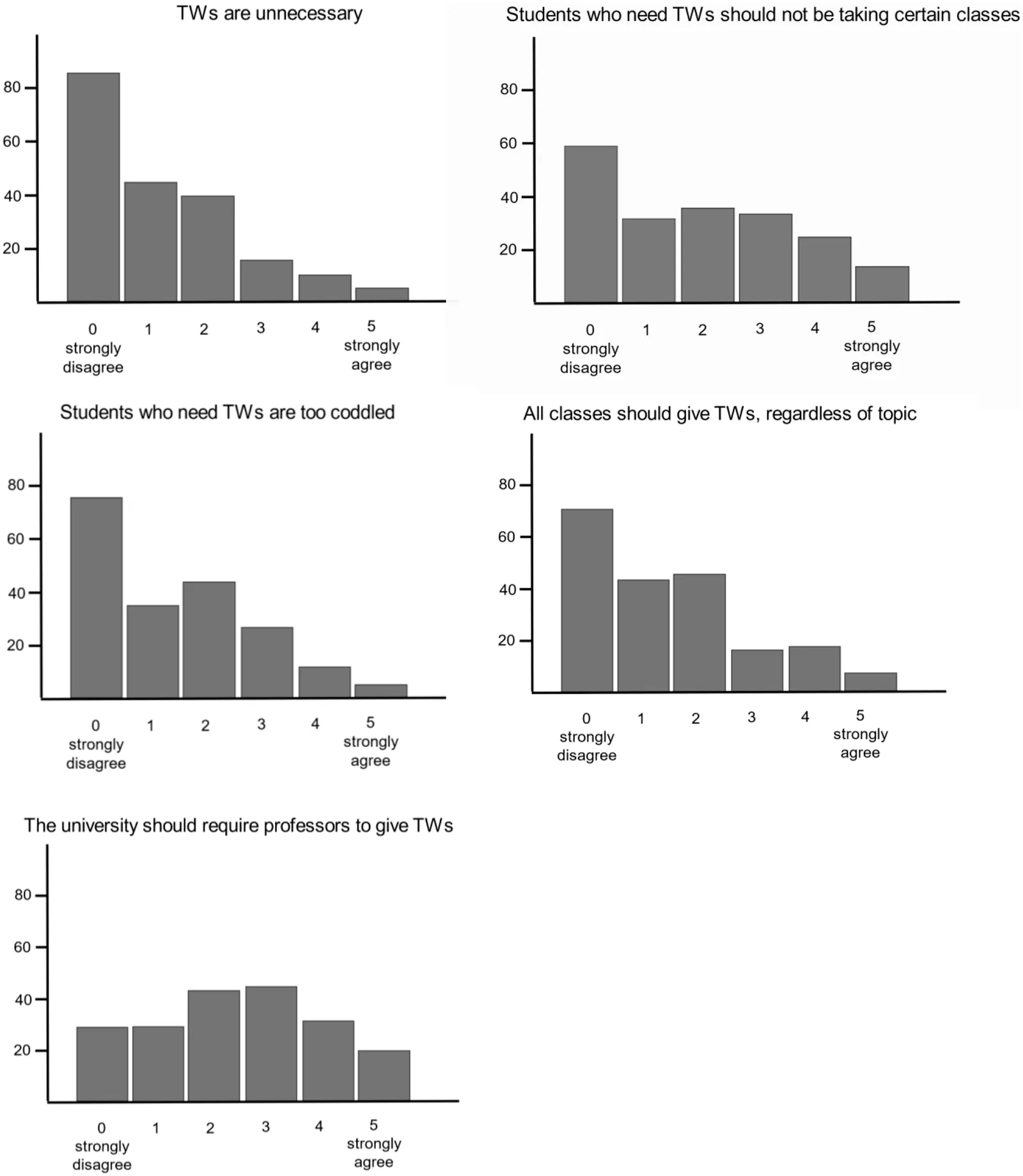
Distribution of responses for the five items of the general attitude towards trigger warnings (TW) scale.

Students’ attitudes toward trigger warnings were significantly above the midpoint of the 0–5 point scale (*M* = 2.88, *SD* = 1.07), *t* (197) = 5.04, *p* < .001, 95% CI [0.23, 0.53], *d* = 0.50, indicating their attitudes were positive. Students significantly *disagreed* that warnings are unnecessary, that students who need warnings should not be taking classes with sensitive or offensive material, and that students who need warnings are coddled. They also significantly disagreed, however, that all classes should give warnings regardless of topic. They neither agreed nor disagreed significantly that professors should be required to use warnings. Figure 1 depicts the distribution of students’ responses on the five items and Table 1 provides detailed statistics. In response to the yes/no question whether warnings should be given before potentially upsetting material, 83% responded “yes” and 17% “no”. See Supplemental Table S2 for students’ responses on the reasons why warnings should be given.

### Beliefs

**Figure 2.**
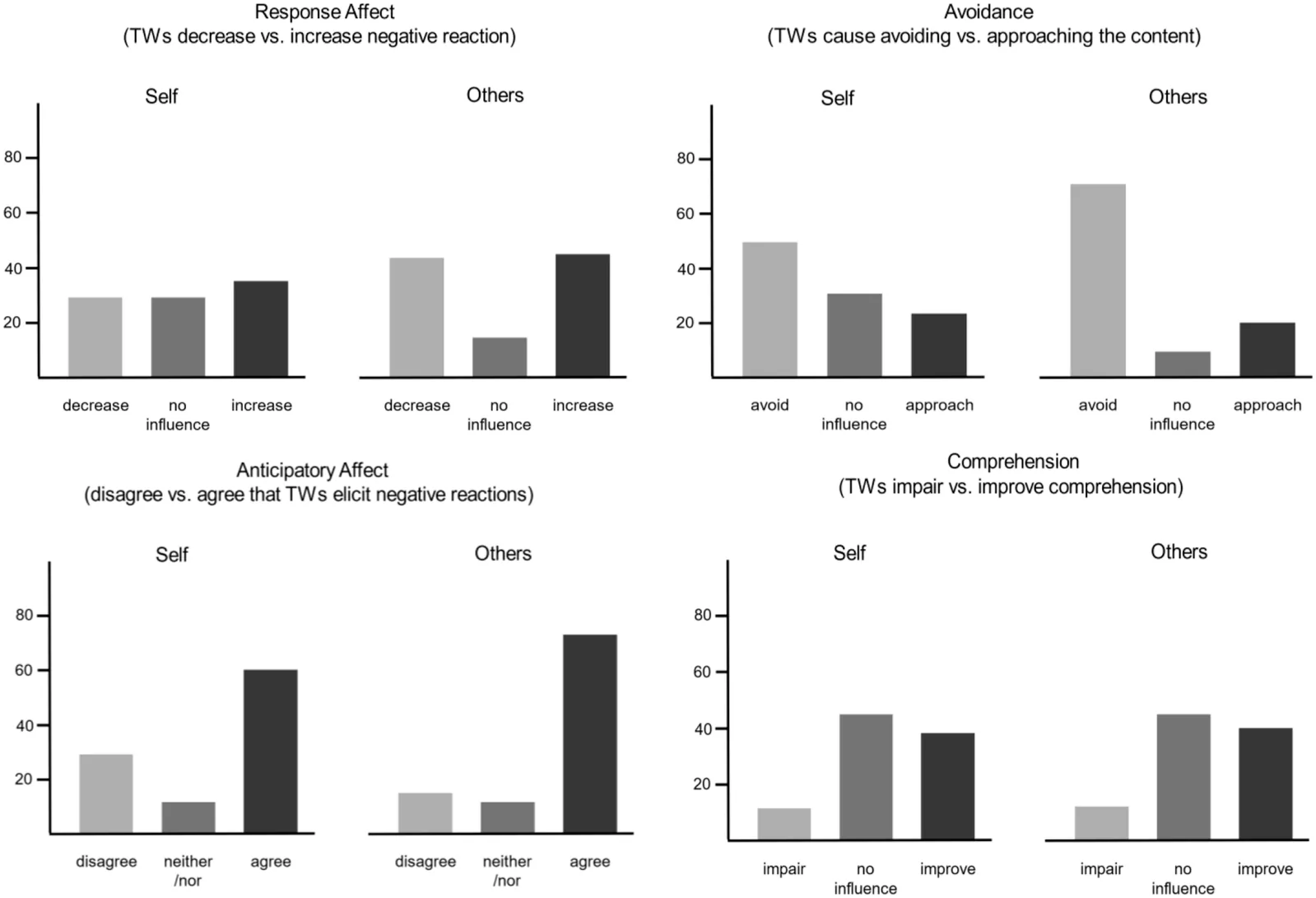
Distribution of responses of the dummy-coded variable beliefs about the effects of trigger warnings (TWs) on self and others in the four domains.

Following our preregistration, we first calculated means for students’ beliefs about the effects of trigger warnings on themselves and others, respectively, in each domain. We then examined whether students’ beliefs significantly differed from the scale midpoint of the bipolar items. Table 2 provides statistics for each item. However, because mean values hide the distribution of the responses, we also dummy-coded the responses into the categories below scale midpoint (−1), at midpoint (0), and above midpoint (1), and descriptively analyzed the categories (Figure 2).

**Table 2.** Ms, SDs, and One-Sample T-Tests on the Scale Midpoint (0) for Each Item on Beliefs About the Effects of Trigger Warnings (TWs) in the Four Domains.

Item	*M* (*SD*)	*df*	*t*	*p*	95% CI	*d*
Response affect (TWs *increase* negative reaction)						
Self	0.08 (1.52)	197	0.75	.454	[−0.13, 0.29]	0.05
Others	−0.04 (1.74)	197	0.29	.775	[−0.28, −0.21]	0.02
Avoidance (TWs cause *approachin*g the content)						
Self	−0.52 (1.60)	197	4.57	<.001	[−0.74, −0.30]	0.33
Others	−0.98 (1.67)	197	8.32	<.001	[−1.22, −0.75]	0.59
Anticipatory affect (TWs elicit *negative* reactions *before* the content)						
Self	0.39 (2.13)	197	2.60	<.010	[0.10, 0.69]	0.19
Others	1.06 (1.67)	197	8.89	<.001	[0.82, 1.29]	0.63
Comprehension (TWs *improve* comprehension)						
Self	0.57 (1.27)	197	6.26	<.001	[0.39, 0.74]	0.46
Others	0.74 (1.27)	197	8.16	<.001	[0.56, 0.92]	0.58

#### Response Affect

On average, students did not significantly believe trigger warnings would change their own or others’ reaction to the material (Table 2). Regarding the dummy-coded response categories, 31% reported believing warnings would dampen their own negative emotional reaction to the material and that of others (42%), 31% believed warnings would have no effect on themselves or others (14%), and 37% believed warnings would increase their negative reaction or that of others (43%; Figure 2).

#### Avoidance

On average, students believed trigger warnings would lead themselves and others to avoid the material. Forty-eight percent believed warnings would lead them to avoid the material (71% for others); 30% believed warnings would have no effect on them (9% for others); 22% believed warnings would cause them to approach the material (20% for others).

#### Anticipatory Affect

On average, students agreed trigger warnings would increase a negative anticipatory reaction of themselves and others. Sixty percent agreed warnings would increase their own negative anticipatory reaction (73% for others); 12% neither agreed nor disagreed (11% for others); 29% disagreed (16% for others).

#### Comprehension

On average, students believed trigger warnings would improve the comprehension of the materials by themselves and others. However, 12% believed warnings would impair their comprehension (13% for others); 44% believed warnings had no effect (34% for others), and 44% believed warnings would improve their comprehension (47% for others).

### Beliefs: Self Versus Other

To explore whether students believed the effects of trigger warnings would be stronger on others than themselves, in each domain, we compared their beliefs about the effect of warnings on themselves with their beliefs about the effect on others using paired-sample T-tests. Beliefs about the response to the material did not significantly differ between others (*M* = −.04, *SD* = 1.74) and self (*M* = .08, *SD* = 1.52), *t* (197) = 1.31, *p* = .191, 95% CI [-0.06, 0.29], *d* = 0.09. Beliefs about avoidance were stronger for others (*M* = −0.98, *SD* = 1.67) than self (*M* = −0.52, *SD* = 1.60), *t* (197) = 3.69, *p* < .001, 95% CI [0.22, 0.71], *d* = 0.26. Beliefs about a negative anticipatory reaction were also stronger for others (*M* = 1.06, *SD* = 1.67) than self (*M* = 0.39, *SD* = 2.13), *t* (197) = 5.20, *p* < .001, 95% CI [-0.91, −0.41], *d* = 0.37. Finally, beliefs about improved comprehension were also stronger for others (*M* = 0.74, *SD* = 1.27) than self (*M* = 0.57, *SD* = 1.27), *t* (197) = 2.65, *p* = .009, 95% CI [-0.30, −0.04], *d* = 0.19.

### Demographics: Gender and Political Orientation

Using participants’ overall index from the General Attitudes Toward Trigger Warnings Scale (Cares et al., 2018), we found that women (*M* = 3.04, *SD* = 0.99) had more positive attitudes toward trigger warnings than men (*M* = 2.52, *SD* = 1.13), *t* (185) = 3.28, *p* = .001, 95% CI [0.21, 0.83], *d* = .50. Moreover, the more left-leaning students’ political orientation was, the more positive attitudes they had, *r* = −.44, *p* < .001.

Women tend to have a more left-leaning political orientation than men (e.g., Langsæther & Knutsen, 2024). This was also the case in our sample (women: *M* = 2.81, *SD* = 1.60; men: *M* = 3.55, *SD* = 2.04), *t* (185) = 2.77, *p* = .006, 95% CI [-1.28, −0.22], *d* = .42. Therefore, to explore whether gender and political orientation were still related to attitudes toward trigger warnings after controlling for each other, we estimated a General Linear Model. Our dependent variable was the attitudes toward trigger warnings index. We entered gender as fixed factor and political orientation as covariate. We observed main effects of gender, *F* (1, 184) = 5.40, *p* = .021, 95% CI [0.05, 0.64], *d* = .34, and political orientation, *F* (1, 184) = 33.79, *p* < .001, 95% CI [-0.31, −0.15], *d* = .86. This pattern indicates that gender and political orientation each significantly contributed to predicting attitudes toward trigger warnings when controlling for each other.

## Discussion

Our study suggests that the use of trigger warnings in academia, originating in the U.S., spread to Germany, a European, non-English-speaking country. A substantial minority of students (40%) had witnessed students demanding warnings. A majority (58%) had warnings at least once during their studies.

Students held rather positive attitudes about warnings. Although on average they rejected the view that warnings should be given regardless of topic, the vast majority (83%) agreed warnings should be given before upsetting material, and almost half (49%) agreed it should be mandatory. Female students and more left-leaning students had more positive attitudes towards trigger warnings. This fits well with the idea that female morality and left-wing morality involves an increased sensitivity to harm and care (Gilligan, 1982; Graham et al., 2009; Haslam, 2016). The political landscape in Germany is similar to the U.S. and UK in that the German political parties can be aligned on a left-right dimension, and the left and right hold similar positions as in the U.S. and UK.

Students’ beliefs about the effects of trigger warnings varied but were somewhat miscalibrated to the actual effects as identified by Bridgland et al. (2023). Although their meta-analysis found no effect of warnings on a reduced negative reaction to or avoidance of the material, a substantial proportion of students believed that warnings would reduce their negative reaction and lead them to avoid the material. A substantial proportion also believed that warnings would improve their comprehension, although the meta-analysis found no evidence for improved comprehension. Finally, most students correctly estimated that warnings could elicit a negative anticipatory reaction even before the material was presented. Perhaps paradoxically, they nevertheless advocated their use.

Beliefs are a powerful driver of behavior (Ajzen, 1991) and shape people’s social reality. Beliefs can also become self-fulfilling prophecies (Rosenthal, 2002). If students inaccurately believe that trigger warnings would protect them from experiencing negative emotions, this may lead them to think they need trigger warnings even when they do not.

Students also believed that the effects of trigger warnings were stronger on others than on themselves. This is consistent with research suggesting that people think others are more vulnerable to negative events than they are (Perloff & Fetzer, 1986). It also fits with evidence that students advocate restrictive measures out of pro-social concern for others (Clark et al., 2023).

### Limitations

Our online convenience sample is not representative of the general German university student population. For example, there were substantially more women than men in our sample (62.7% women, 37.3% men), whereas the gender distribution in the general German university student population is more balanced (51.6% women, 48.4% men; Federal Statistics Office of Germany, 2024). However, our gender distribution is not atypical for the subjects in which trigger warnings are most likely to occur (psychology, humanities). Future work should examine the use of trigger warnings in non-Western countries (Henrich et al., 2010).

## Conclusion

Students were positively inclined to the use of trigger warnings. However, because students’ beliefs about the effects of warnings did not match well with the actual effects, our findings highlight the need to make students (and lecturers) aware of the empirical evidence on the effects of trigger warnings.

## Supplemental Material

Supplemental Material - Students’ Beliefs About Trigger Warnings Supplemental Material for Students’ Beliefs About Trigger Warnings by A. Timur Sevincer, Leonie Tenbrueggen, and Marvin Sokolis in Psychological Reports

## Data Availability

The data, analysis code, materials, and preregistration pertaining to this article are available at: https://shorturl.at/L8zT6.

## Note

1. The two omitted items were: “Other classes that cover victimization material should have trigger warnings in the syllabus”, and “Other classes that cover victimization material should have trigger warnings given verbally in class”.
