# Supplementary material for: Students’ Beliefs About Trigger Warnings
Source: Psychol Rep. 2024 Dec 13;129(5):4189–201. doi: 10.1177/00332941241308788 (PMC13530343; doi:10.1177/00332941241308788)
Supplement: Supplementary file 1 [file sj-pdf-1-prx-10.1177_00332941241308788.pdf]

Supplementary Material

Supplementary Table S1

Frequencies of participants' major subjects.

Subject	*n*	%
Psychology	45	22
Languages	21	10
Education/Communication	12	6
Law	18	9
Political Sciences/History	10	5
Sociology/Social Work	12	10
Economics/Informatics	14	7
Other	62	30
No Response	11	5
Total	205	100

Supplementary Table S2

Trigger Warnings Attitudes Assessment: Absolute numbers and percentages of the 164 students who responded with "yes" that trigger warnings should be given who agreed to each of the six reasons why trigger warnings should be given.

Item	*n*	%
Trigger warnings help to protect vulnerable populations (such as people who have posttraumatic stress disorder) from psychological harm.	135	82
It's not fair that vulnerable people, such as those with posttraumatic stress disorder, should be exposed to material that causes them distress without a warning.	88	54
Presenting material that is offensive to a specific group of people (racist or sexist material, for example) can cause psychological harm to members of that group.	79	48
It's not fair to present material that is offensive to a specific group of people (material that is racist or sexist, for example).	79	48
Offensive material can cause psychological harm to anyone.	79	48
It's not fair to present material that is offensive to anyone without giving a warning first.	76	47
